# Mismatch Repair Deficiency as a Predictive Biomarker for Immunotherapy Efficacy

**DOI:** 10.1155/2017/4719194

**Published:** 2017-07-10

**Authors:** Giulia Viale, Dario Trapani, Giuseppe Curigliano

**Affiliations:** Division of Early Drug Development, European Institute of Oncology, Via Ripamonti 435, Milan, Italy

## Abstract

Immunotherapy has revolutionized cancer treatment. Immune-checkpoint inhibitors, on balance, showed a favorable efficacy/toxicity profile with durable response in different cancer types. No predictive biomarker has been validated thus far to select patients who would benefit from therapy. Among the candidate predictive biomarkers, mismatch repair status of the tumor is currently one of the most promising. Indeed, tumors displaying mismatch repair deficiency or microsatellite instability showed remarkable response to immunotherapy in clinical trials. This correlation has been first reported in colorectal cancers, but similar results have been observed also in other cancer types. The possible mechanism behind this correlation may be the higher mutational load observed in mismatch repair deficient tumors, leading to neoantigens formation, recruitment of immune cells, and release of proinflammatory factors in the microenvironment. These results support an approach to treatment based on assessment of the genomic stability of the tumor besides its biologic characteristics and may change our therapeutic decision making process. However, due to the small percentage of patients with tumors displaying mismatch repair deficiency, data from clinical trials should not be considered definitive and need further confirmation.

## 1. Introduction

### 1.1. Immunotherapy and Immune-Checkpoint Inhibitors

The immune system manipulation has been increasingly acquiring a central role in cancer treatment; thanks to a deeper understanding of immune system function in terms of antitumor activity, several strategies targeting immune cells and the microenvironment are under development. Undoubtedly, immune-checkpoint molecules are some of the best-characterized and studied mechanism of interaction between immune system and cancer.

Cytotoxic T-lymphocyte-associated antigen 4 (CTLA-4) has been the first immune-checkpoint molecule to be clinically targeted. Its main role is to regulate T cells activation at the time of their initial response to the antigen, counterbalancing the effect of T cell receptor (TCR)/CD3 activating and CD28 costimulation signals. CTLA4 function is exerted by binding to its ligand, CD80/CD86 (mainly expressed by the antigen presenting cells, APCs), thus blocking the costimulation signals of T cells and dampening the amplitude of the response, resulting in immune suppression [1].

Similarly, a well-characterized immune-checkpoint molecule is the programmed cell death protein 1 (PD-1), expressed by activated T cells, B cells, and natural killer (NK); PD-1 regulates the inflammatory responses mainly in the peripheral tissues, limiting collateral tissue damage in inflammatory process resolution and autoimmunity phenomena [1, 2]. PD-1 activity is modulated by a specific set of ligands, the programmed death-ligand 1 (PD-L1) and programmed death-ligand 2 (PD-L2).

Inflammatory signals (i.e., interferon-*γ*, IFN*γ*) in the microenvironment induce expression of PD-L1 and PD-L2. PD-L1 is the most characterized PD-1 partner; it is commonly expressed on T helper cells, myeloid derived suppressor cells in the tumor microenvironment, and cancer cells, too.

The activity of PD-1/PD-L1 axis is immunosuppressive: in particular, excessive induction of PD-1 pathway in the setting of chronic antigen exposure as well as in cancer has been shown to induce an exhausted or anergic phenotype in T cells [1, 2], thus impairing the antitumor activity of the immune system.

Reverting immunosuppression promoted by immune-checkpoint molecules like CTLA4, PD-1, and PD-L1 was demonstrated to be an effective anticancer therapeutic strategy. Immune-checkpoint inhibitors have shown a remarkable clinical efficacy and durable response with a favorable toxicity profile in a large number of solid and hematologic malignancies [3], such as melanoma [4, 5], lung cancer [NSCLC, [6, 7]], bladder cancer [8], and renal cancer [RCC, [9]].

In particular, Ipilimumab, a CTLA4 blocking monoclonal antibody, has been FDA-approved for the treatment of metastatic melanoma (MM), after showing an overall survival advantage with a favorable toxicity profile. Another anti-CTLA4, Tremelimumab, has been more recently developed and received orphan drug status for the treatment of malignant mesothelioma. Similarly, FDA granted accelerated approval of PD-1 inhibitors Nivolumab and Pembrolizumab for the treatment of different tumors (as MM, advanced NSCLC, head and neck squamous cell carcinoma, and classical Hodgkin's lymphoma). On the other hand, anti-PD-L1 antibodies Atezolizumab, Avelumab, and Durvalumab obtained as well FDA accelerated approval in different solid tumors, as advanced urothelial bladder cancer and Merkel cells carcinoma. Many other anti-PD-1/PD-L1 molecules are being developed with promising results in clinical trials.

### 1.2. Predictive Biomarkers

No predictive biomarkers have been validated thus far to select patients who would mostly benefit from immunotherapy, sparing nonresponders from the risk of severe adverse events and saving costs.

PD-L1 protein expression by tumor and immune cells has been investigated as a potential predictive biomarker [10], but its correlation with immunotherapy efficacy is still debated [11–13] and technical issues prevent its routine use in clinical practice [6, 8]. In addition, PD-L1 expression varies widely between tumor types and presents a significant intrapatient heterogeneity with a frequent discordance between primary tumors and metastases [14, 15]. Probably, PD-1/PD-L1 expression reflects a dynamic process influenced by multifactorial events like concomitant treatments, mainly targeted therapies [16]. Other promising candidate predictive biomarkers are currently under investigation [17], particularly cells or molecules related to immune response in tumor microenvironment such as tumor infiltrating lymphocytes (TILs) [18], indoleamine 2,3-dioxygenase (IDO) [19], BCL-2 interacting mediator of cell death-Bim [20], and interferon-gamma [21].

A different possible approach to predict immunotherapy efficacy is to analyze the somatic mutational landscape of the tumor, since a high mutational burden has been shown to correlate with benefit from immunotherapy [22, 23]. However, whole exome sequencing is time and cost consuming and currently not feasible routinely [24].

An increased rate of somatic mutations has been observed particularly in mismatch repair (MMR) deficient tumors that indeed have shown responsiveness to immunotherapy independently of histologic and anatomic defined subtypes [25]. Thus, MMR status of the tumor may represent a potentially feasible and useful predictive biomarker; besides, it has a well-known prognostic role. Although MMR deficient cancers frequently show aggressive histological features like high nuclear grade at microscopy, they have a paradoxically favorable outcome. In a large series of young colorectal cancer patients, microsatellite instability was associated with a significant survival advantage independently of all standard prognostic factors, including tumor stage [26].

## 2. Mismatch Repair: Role and Implications

MMR system is a DNA integrity maintenance system. The main role of MMR proteins is the correction of single base nucleotide mismatches (insertions or deletions) generated during DNA replication and recombination, thus maintaining the genomic stability [27]. These proteins are responsible for the corrections of mismatches that occurred during meiosis and mitosis [28] and might have a potential role in oxidative DNA damage repair [29] as well as in antibody class-switch recombination [30].

The mechanism of MMR involves at least three different processes: recognition, excision, and resynthesis. Recognition of single base replication errors is performed by the MutS*α* (MSH2-MSH6 heteroduplex) or MutS*β* (MSH2-MSH3 heteroduplex), excision of the lagging strand from the mismatch by one of the MutL complexes (mainly MutL*α* formed by MLH1/PMS2) recruited by MutS protein, and resynthesis of the excised-DNA and ligation by DNA polymerase delta and DNA ligase I [31].

Loss of expression of one of the MMR proteins may result from inherited germline defects (usually mutations) in one of the mismatch repair genes; rarely both of inherited alleles are mutated as in constitutional MMR deficiency syndrome leading to cancer in early childhood called constitutional mismatch repair deficiency [32]. More frequently, only one mutated allele is inherited and loss of the other allele occurs somatically, as in Lynch syndrome (LS), an autosomal dominant condition that predisposes to cancer development (particularly colorectal cancer (CRCs) and ovarian and endometrial cancer) [28]. Alternatively, MMR deficiency may be derived by either somatic mutation or methylation of one of the MMR genes: sporadic MMR deficient tumors are often the result of epigenetic silencing of MLH1 promoter due to a hypermethylation mechanism [33, 34].

Due to its role in genomic stability, MMR deficiency leads to accumulation of somatic mutations [31]. Microsatellites—repetitive short (1–6 base pairs) tandem DNA sequences scattered throughout the whole genome—are particularly subject to copying errors when mismatch repair is compromised. Therefore, it is possible to trace the MMR deficiency by studying the microsatellites: when they are demonstrated to be hypermutated (instable), MMR may be deducted.

Recent evidence showed that tumors with microsatellite instability due to MMR deficiency have different phenotype and histologic characteristics—and in some cases even a different prognosis [35]—as compared to MMR proficient tumors [36–38].

MMR status of the tumor may be assessed either by immunohistochemistry (IHC) that tests loss of a MMR protein or by PCR based assays for microsatellite instability [39]. IHC and MSI testing are complementary as both have a false negative rate of approximately 5–10%.

## 3. MMR Status as a Predictor of Immunotherapy Efficacy: Clinical Data

The correlation between tumor MMR status and the outcome in patients treated with immunotherapy has been initially observed in CRCs treated with PD-1 blocker: only 1 of 33 patients with CRCs showed a response to immune-treatment, despite remarkable efficacy of these anticancer agents in other tumor subtypes [40, 41]. Since both MMR deficiency and immunotherapy benefit are expected in a very small fraction of CRCs patients, a possible correlation between the two has been hypothesized and confirmed in a recent phase II study [25]. A total of 41 patients with treatment refractory progressive MMR deficient and proficient metastatic CRCs were recruited, as well as a small proportion of patients with MMR deficient cancers of other types (cholangiocarcinoma, endometrial, small bowel, and gastric cancer). The three different cohorts, consisting of 11, 21, and 9 patients, respectively, were treated with Pembrolizumab. An immune related objective response rate (ORR) of 40% was observed in MMR deficient CRCs compared to a total lack of response in MMR proficient CRCs (ORR 0%), with a similar difference in progression free survival (PFS) rate at 20 weeks between the two groups (78% versus 11%). Likewise, MMR deficient tumors other than CRCs showed an ORR of 71% with a PFS of 67%. The difference in survival of patients with MMR deficient and proficient CCRs is independent from other prognostic factors, since no significant differences in PFS between the two groups were observed while receiving previous chemotherapy regimens. Interestingly, all the six patients with MMR deficient tumors not associated with Lynch syndrome had an objective response, whereas only 27% of patients with Lynch syndrome had a response. However—due to the small sample size of the study population—these results require further confirmation.

Numerous studies demonstrated that MMR status correlates also with chemotherapy resistance, with MMR deficient tumors being commonly resistant to methylating agents, platinum compounds and fluoropyrimidines [42, 43]. A possible explanation may involve DNA damage response proteins (i.e., ataxia telangiectasia mutated (ATM) and ataxia telangiectasia and Rad3-related protein (ATR)), recruited by MMR proteins during treatment with DNA-damaging agents. ATM/ATR, in turn, lead to cell cycle arrest, DNA repair, or apoptosis through DNA damage checkpoint proteins activation [43, 44]. MMR deficiency might alter this mechanism and confer resistance to many chemotherapies [45].

## 4. Exploiting Mismatch Repair Deficiency as a Predictor of Immunotherapy Efficacy: Biologic Rationale

Multiple possible mechanisms have been proposed to explain the correlation between MMR deficiency and immune response in some cancer types. It has been observed that MMR deficiency is associated with a 10–100-fold-increased rate of somatic mutations [46]. The genomic analysis of whole exome sequences of primary tumor samples from 15 patients included in the study by Le and colleagues [25] revealed a mean of 1782 somatic mutations per tumor in MMR deficient neoplasms, compared to 73 mutations per tumor in MMR proficient ones.

MMR deficiency may provide an upregulation of a large number of genes involved in the immune response, as proinflammatory cytokines and cytotoxic mediators through a genome expression dysregulation, thus resulting in an increased secretion of soluble mediators in the tumor microenvironment with the subsequent activation of the PD-1 pathway. This might justify the observation that MMR deficient tumors are immunogenic [47].

In addition, somatic mutations may lead to the expression of a high number of tumor neoantigens that could promote the release of proinflammatory cytokines and elicit the recruitment and activity of cytotoxic T cells [48, 49]. Indeed, it has been described that MMR deficient tumors have a dense infiltration of intraepithelial CD8+ T lymphocytes and activated T helper cells.

Nevertheless, a recent study reported that the active antitumor immune microenvironment may be counterbalanced by the presence of immune-checkpoint ligands (i.e., PD-1/PD-L1, CTLA4, LAG3, IDO1, TIM3, GITR, and TIGIT) that favor immune escape, thus suggesting that TILs are mainly directed at neoantigens [50]. This hypothesis appears to be confirmed by clinical data, as NSCLC and MM—cancers known to have a high mutational load as a result to exposure to cigarette smoking and UVA radiation, respectively—are among the tumor types most responsive to PD-1 blockade. Moreover, patients affected by NSCLC with a high number of somatic mutations have significantly better clinical outcomes compared to patients with less mutated tumors [51]. A similar correlation has been observed for MM patients treated with anti-CTLA-4 therapy [52]. Since neoantigens are frequently different between patients and a single mutation cannot predict response to immunotherapy, the candidate predictive factor is the presence of high mutational load and the consequent recruitment of T cells in the microenvironment [53].

A recent study evaluated PD-L1 expression in MMR deficient endometrial tumors, either Lynch syndrome associated or sporadic tumors (with MLH1 hypermethylation), and showed a significant higher PD-L1 expression compared to the MMR proficient counterpart [54]. Likewise, a case of MMR deficient sporadic high-grade urothelial carcinoma of the renal pelvis treated with immunotherapy was reported: the patient experienced a prolonged complete remission in two months [55]. Besides sporadic case reports, the landscape of microsatellite instability across different cancer subtypes is still poorly understood. A recent study examined 5.930 cancer exomes from 18 cancer types at more than 200.000 microsatellite loci, analyzing also cancer types for which MSI status has not been previously tested in clinical practice. The average number of unstable sites varied considerably by cancer type, ranging from a minimum of 765 unstable sites in thyroid carcinomas, to a maximum of 2.315 in colon cancers [56]. Endometrial, colon, and gastric cancer were confirmed to have the highest proportion of microsatellite instability; however most cancer types examined (14 of 18) included one or more representatives with microsatellite instability, suggesting that this could be a generalized, continuous rather than discrete, cancer phenotype. This heterogeneity adds further complexity to the scenario of potential predictive biomarkers of immunotherapy response. Interestingly, this analysis identified loci more likely to be unstable in specific cancer types, resulting in specific signatures in cancer-associated genes, suggesting that instability patterns may reflect selective pressures and can potentially identify novel cancer drivers [56].

## 5. Conclusion

Data from recent clinical studies suggest that immunotherapy with immune-checkpoint inhibitors may represent a promising therapeutic strategy for patients with MMR deficient tumors, independently of subtype. The proportion of candidate patients, however, is relatively small, because MMR deficiency has been observed only in about 4% of metastatic CRCs, 11% of ovarian carcinomas, 18% of endometrial cancers, and 1% of pancreatic cancers [25, 57]. A few reports showed promising results also in cancers not usually treated with immunotherapy, thus suggesting that screening for MMR deficiency should be potentially offered to all patients with advanced disease, independently from histology. Accordingly, some current ongoing studies are exploring the potential predictive role of MMR status, as summarized in Table 1. Most importantly, these results support an approach to treatment based on genetic status of tumor regardless of cancer subtype. Eventually, a better understanding of pathologic and genomic features of MMR deficient tumors may allow the identification of other biomarkers (such as TILs, immune-checkpoint proteins, and genomic mutations) potentially useful in clinical routine practice to predict response to immunotherapy or as surrogate markers of early response to therapy [17]. Indeed, microsatellite instability alone may not be sufficient to predict response to immune-checkpoint inhibitors, as, for example, not all tumor neoantigens may bind the major histocompatibility complex (MHC) class I. Additional immune-regulatory mechanisms may have a role as a contributor of anti-PD-1/PD-L1 response, as T cell absence and genetic/epigenetic alterations [58]. Accordingly, it has been demonstrated that PI3K/PTEN/AKT pathway hyperactivity may dampen antitumor immune activation when PTEN-null tumors are exposed to an immune-checkpoint inhibitor, thus suggesting a specific genetic regulatory mechanism [59].

A global concept has recently been summarized by Chen and Mellman [60] in the definition of an “immune set-point” as a global immune activation status potentially predictive of response to immune therapies as well as a tool to guide the choice of different strategies of treatment.

## Figures and Tables

**Table 1:** Ongoing clinical trial with immune-checkpoint inhibitors alone or in a combination regimen according to mismatch repair status for different solid tumors. Last updated, April 2017.

Experimental arm	Active comparator regimen	Disease	Setting	Phase	Comments	ClinicalTrials.gov Identifier
Atezolizumab + FOLFOX	FOLFOX	CRC	Adjuvant, stage III	3	CT plus IO up to 25 courses	NCT02912559

Pembrolizumab	FOLFOX FOLFIRI Bevacizumab Cetuximab	CRC	IV	3	KEYNOTE-177 IO for up to 35 treatments	NCT02563002

*GVAX*° Pembrolizumab Cyclophosphamide	Single arm	CRC	Advanced	2	MMRp	NCT02981524

*AZD9150* ^§^ Durvalumab	Single arm	Pancreatic, NSCLC, and MMRd CRC	Advanced	2	—	NCT02983578

Pembrolizumab Poly-ICLC^+^	Single arm	MMRp CRC	IV	1/2	IO for 1 year	NCT02834052

DS-8273^∧^ Nivolumab	NA	MMRp CRC	IV	1	—	NCT02991196

Nivolumab	Single arm	Hypermutated malignancies	Recurrent or refractory disease	1/2	Pediatric patients (12 months to 18 years of age) Biallelic MMRd^*∗*^	NCT02992964

Nivolumab	Single arm	mCRPC with mutations in DNA repair defects^#^	IV	2	ImmunoProst Trial IO until progression or unacceptable toxicity	NCT03040791

Avelumab Ad-CEA vaccine Standard of care	FOLFOX Bevacizumab Capecitabine	CRC	IV	2	CT and IO with maintenance	NCT03050814

Pembrolizumab	Single arm	High-grade gliomas, diffuse intrinsic pontine gliomas, or hypermutated brain tumors	NA	2	IO for 34 courses	NCT02359565

FOLFOX: Fluorouracil, Leucovorin, and Oxaliplatin combination regimen. CRC: colorectal cancer. CT: chemotherapy. IO: immunotherapy. FOLFIRI: Fluorouracil, Leucovorin, and Irinotecan. MMRp: mismatch repair proficient profile. MMPd: mismatch repair deficient profile. NSCLC: non-small cell lung carcinoma. mCRPC: metastatic castration-resistant prostate cancer; °GVAX, cancer vaccine composed of irradiated tumor cells genetically modified to secrete granulocyte-macrophage colony-stimulating factor; ^§^AZD9150, antisense oligonucleotide inhibitor of STAT3; ^+^Poly-ICLC (carboxymethylcellulose, polyinosinic-polycytidylic acid, and poly-L-lysine double-stranded RNA), ligand of TLR3; ^∧^DS-8273a, anti-human death receptor 5 (DR5) agonistic antibody; ^*∗*^patients must have evidence of biallelic mismatch repair deficiency either in their tumor tissue (by immunohistochemistry or sequencing) or in their germline (by sequencing) and/or evidence of hypermutant malignancy by whole exome sequencing with a mutation load > 100 per exome; ^#^the germline and somatic DRD (BRCA1, BRCA2, ATM, PTEN, CHEK2, RAD51C, RAD51D, PALB2, MLH1, MSH2, MSH6, and PMS2) will be assessed by T-NGS of metastatic sites or by liquid biopsy.

## References

[1] Pardoll D. M. (2012). The blockade of immune checkpoints in cancer immunotherapy. *Nature Reviews Cancer*.

[2] Moreno B. H., Ribas A. (2015). Anti-programmed cell death protein-1/ligand-1 therapy in different cancers. *British Journal of Cancer*.

[3] Ansell S. M., Lesokhin A. M., Borrello I. (2015). PD-1 blockade with nivolumab in relapsed or refractory Hodgkin's lymphoma. *The New England Journal of Medicine*.

[4] Robert C., Long G. V., Brady B. (2015). Nivolumab in previously untreated melanoma without BRAF mutation. *The New England Journal of Medicine*.

[5] Weber J. S., D'Angelo S. P., Minor D. (2015). Nivolumab versus chemotherapy in patients with advanced melanoma who progressed after anti-CTLA-4 treatment (CheckMate 037): a randomised, controlled, open-label, phase 3 trial. *The Lancet Oncology*.

[6] Garon E. B., Rizvi N. A., Hui R. (2015). Pembrolizumab for the treatment of non-small-cell lung cancer. *The New England Journal of Medicine*.

[7] Brahmer J., Reckamp K. L., Baas P. (2015). Nivolumab versus docetaxel in advanced squamous-cell non-small-cell lung cancer. *The New England Journal of Medicine*.

[8] Powles T., Eder J. P., Fine G. D. (2014). MPDL3280A (anti-PD-L1) treatment leads to clinical activity in metastatic bladder cancer. *Nature*.

[9] Motzer R. J., Escudier B., McDermott D. F. (2015). Nivolumab versus everolimus in advanced renal-cell carcinoma. *New England Journal of Medicine*.

[10] Taube J. M., Klein A., Brahmer J. R. (2014). Association of PD-1, PD-1 ligands, and other features of the tumor immune microenvironment with response to anti-PD-1 therapy. *Clinical Cancer Research*.

[11] Kefford R., Ribas A., Hamid O. (2014). Clinical efficacy and correlation with tumor PD-L1 expression in patients with melanoma treated with the anti-PD-1 monoclonal antibody MK-3475. *Journal of Clinical Oncology*.

[12] Carbognin L., Pilotto S., Milella M. (2015). Differential activity of nivolumab, pembrolizumab and MPDL3280A according to the tumor expression of programmed death-ligand-1 (PD-L1): sensitivity analysis of trials in melanoma, lung and genitourinary cancers. *PLoS ONE*.

[13] Muro K., Bang Y., Shankaran V. (2015). Relationship between PD-L1 expression and clinical outcomes in patients (Pts) with advanced gastric cancer treated with the anti-PD-1 monoclonal antibody pembrolizumab (Pembro; MK-3475) in KEYNOTE-012. *Journal of Clinical Oncology*.

[14] Madore J., Vilain R. E., Menzies A. M. (2015). PD-L1 expression in melanoma shows marked heterogeneity within and between patients: Implications for anti-PD-1/PD-L1 clinical trials. *Pigment Cell and Melanoma Research*.

[15] Mitchell P., Murone C., Asadi K. (2015). PD-L1 expression in NSCLC: analysis of a large early stage cohort: and concordance of expression in primary, node and metastasis. *Journal of Thoracic Oncology*.

[16] Gainor J. F., Sequist L. V., Shaw A. T. (2015). Clinical correlation and frequency of programmed death ligand-1 (PD-L1) expression in EGFR-mutant and ALK-rearranged non-small cell lung cancer (NSCLC). *Journal of Clinical Oncology*.

[17] Gibney G. T., Weiner L. M., Atkins M. B. (2016). Predictive biomarkers for checkpoint inhibitor-based immunotherapy. *The Lancet Oncology*.

[18] Tumeh P. C., Harview C. L., Yearley J. H. (2014). PD-1 blockade induces responses by inhibiting adaptive immune resistance. *Nature*.

[19] Hamid O., Schmidt H., Nissan A. (2011). A prospective phase II trial exploring the association between tumor microenvironment biomarkers and clinical activity of ipilimumab in advanced melanoma. *Journal of Translational Medicine*.

[20] Dronca R. S., Markovic S., Kottschade L. A. (2015). Bim as a predictive T-cell biomarker for response to anti-PD-1 therapy in metastatic melanoma (MM). *Journal of Clinical Oncology*.

[21] Ferrucci P. F., Gandini S., Battaglia A. (2015). Baseline neutrophil-to-lymphocyte ratio is associated with outcome of ipilimumab-treated metastatic melanoma patients. *British Journal of Cancer*.

[22] Boussiotis V. A. (2014). Somatic mutations and immunotherapy outcome with CTLA-4 blockade in melanoma. *New England Journal of Medicine*.

[23] Rizvi N. A., Hellmann M. D., Snyder A. (2015). Mutational landscape determines sensitivity to PD-1 blockade in non–small cell lung cancer. *Science*.

[24] Watson I. R., Takahashi K., Futreal P. A., Chin L. (2013). Emerging patterns of somatic mutations in cancer. *Nature Reviews Genetics*.

[25] Le D. T., Uram J. N., Wang H. (2015). PD-1 blockade in tumors with mismatch repair deficiency. *Journal of Clinical Oncology*.

[26] Gryfe R., Kim H., Hsieh E. T. K. (2000). Tumor microsatellite instability and clinical outcome in young patients with colorectal cancer. *The New England Journal of Medicine*.

[27] Modrich P. (2006). Mechanisms in eukaryotic mismatch repair. *Journal of Biological Chemistry*.

[28] Peltomäki P. (2005). Lynch syndrome genes. *Familial Cancer*.

[29] Brierley D. J., Martin S. A. (2013). Oxidative stress and the DNA mismatch repair pathway. *Antioxidants and Redox Signaling*.

[30] Smith J. A., Bannister L. A., Bhattacharjee V., Wang Y., Waldman B. C., Waldman A. S. (2007). Accurate homologous recombination is a prominent double-strand break repair pathway in mammalian chromosomes and is modulated by mismatch repair protein Msh2. *Molecular and Cellular Biology*.

[31] Hsieh P., Yamane K. (2008). DNA mismatch repair: molecular mechanism, cancer, and ageing. *Mechanisms of Ageing and Development*.

[32] Wang Q., Lasset C., Desseigne F. (1999). Neurofibromatosis and early onset of cancers in hMLH1-deficient children. *Cancer Research*.

[33] Beggs A. D., Domingo E., Abulafi M., Hodgson S. V., Tomlinson I. P. M. (2013). A study of genomic instability in early preneoplastic colonic lesions. *Oncogene*.

[34] Funkhouser W. K., Lubin I. M., Monzon F. A. (2012). Relevance, Pathogenesis, and testing algorithm for mismatch repair-defective colorectal carcinomas: a report of the association for molecular pathology. *Journal of Molecular Diagnostics*.

[35] Popat S., Hubner R., Houlston R. S. (2004). A meta-analysis of microsatellite instability and colorectal cancer prognosis. *Journal of Clinical Oncology*.

[36] Devaud N., Gallinger S. (2013). Chemotherapy of MMR-deficient colorectal cancer. *Familial Cancer*.

[37] Smyrk T. C., Watson P., Kaul K., Lynch H. T. (2001). Tumor-infiltrating lymphocytes are a marker for microsatellite instability in colorectal carcinoma. *Cancer*.

[38] Haraldsdottir S., Hampel H., Wu C. (2016). Patients with colorectal cancer associated with Lynch syndrome and MLH1 promoter hypermethylation have similar prognoses. *Genetics in Medicine*.

[39] Berg K. D., Glaser C. L., Thompson R. E., Hamilton S. R., Griffin C. A., Eshleman J. R. (2000). Detection of microsatellite instability by fluorescence multiplex polymerase chain reaction. *The Journal of Molecular Diagnostics*.

[40] Topalian S. L., Hodi F. S., Brahmer J. R. (2012). Safety, activity, and immune correlates of anti-PD-1 antibody in cancer. *New England Journal of Medicine*.

[41] Brahmer J. R., Drake C. G., Wollner I. (2010). Phase I study of single-agent anti-programmed death-1 (MDX-1106) in refractory solid tumors: safety, clinical activity, pharmacodynamics, and immunologic correlates. *Journal of Clinical Oncology*.

[42] Stojic L., Brun R., Jiricny J. (2004). Mismatch repair and DNA damage signalling. *DNA Repair*.

[43] Hewish M., Lord C. J., Martin S. A., Cunningham D., Ashworth A. (2010). Mismatch repair deficient colorectal cancer in the era of personalized treatment. *Nature Reviews Clinical Oncology*.

[44] Roos W. P., Kaina B. (2013). DNA damage-induced cell death: From specific DNA lesions to the DNA damage response and apoptosis. *Cancer Letters*.

[45] Meyers M., Hwang A., Wagner M. W. (2003). A role for DNA mismatch repair in sensing and responding to fluoropyrimidine damage. *Oncogene*.

[46] Timmermann B., Kerick M., Roehr C. (2010). Somatic mutation profiles of MSI and MSS colorectal cancer identified by whole exome next generation sequencing and bioinformatics analysis. *PLoS ONE*.

[47] Nishimura H., Nose M., Hiai H., Minato N., Honjo T. (1999). Development of lupus-like autoimmune diseases by disruption of the PD-1 gene encoding an ITIM motif-carrying immunoreceptor. *Immunity*.

[48] Sæterdal I., Bjørheim J., Lislerud K. (2001). Frameshift-mutation-derived peptides as tumor-specific antigens in inherited and spontaneous colorectal cancer. *Proceedings of the National Academy of Sciences of the United States of America*.

[49] Boissière-Michot F., Lazennec G., Frugierz H. (2014). Characterization of an adaptive immune response in microsatellite-instable colorectal cancer. *OncoImmunology*.

[50] Llosa N. J., Cruise M., Tam A. (2015). The vigorous immune microenvironment of microsatellite instable colon cancer is balanced by multiple counter-inhibitory checkpoints. *Cancer Discovery*.

[51] Rizvi N. A., Hellmann A., Kvistborg P. (2015). Mutational landscape determines sensitivity to PD-1 blockade in non-small cell lung cancer. *Science*.

[52] Snyder A., Makarov V., Merghoub T. (2014). Genetic basis for clinical response to CTLA-4 blockade in melanoma. *The New England Journal of Medicine*.

[53] Koster B. D., De Gruijl T. D., Van Den Eertwegh A. J. M. (2015). Recent developments and future challenges in immune checkpoint inhibitory cancer treatme. *Current Opinion in Oncology*.

[54] Sloan E. A., Ring K. L., Willis B. C., Modesitt S. C., Mills A. M. (2017). PD-L1 Expression in Mismatch Repair-deficient Endometrial Carcinomas, Including Lynch Syndrome-associated and MLH1 Promoter Hypermethylated Tumors. *American Journal of Surgical Pathology*.

[55] Castro M. P., Goldstein N. (2015). Mismatch repair deficiency associated with complete remission to combination programmed cell death ligand immune therapy in a patient with sporadic urothelial carcinoma: Immunotheranostic considerations. *Journal for ImmunoTherapy of Cancer*.

[56] Hause R. J., Pritchard C. C., Shendure J., Salipante S. J. (2016). Classification and characterization of microsatellite instability across 18 cancer types. *Nature Medicine*.

[57] Dudley J. C., Lin M., Le D. T., Eshleman J. R. (2016). Microsatellite instability as a biomarker for PD-1 Blockade. *Clinical Cancer Research*.

[58] Yarchoan M., Johnson B. A., Lutz E. R., Laheru D. A., Jaffee E. M. (2017). Targeting neoantigens to augment antitumour immunity. *Nature Reviews Cancer*.

[59] Rizvi N. A., Chan T. A. (2016). Immunotherapy and oncogenic pathways: the PTEN connection. *Cancer Discovery*.

[60] Chen D. S., Mellman I. (2017). Elements of cancer immunity and the cancer-immune set point. *Nature*.

